# The antiviral drug tenofovir, an inhibitor of Pannexin-1-mediated ATP release, prevents liver and skin fibrosis by downregulating adenosine levels in the liver and skin

**DOI:** 10.1371/journal.pone.0188135

**Published:** 2017-11-16

**Authors:** Jessica L. Feig, Aranzazu Mediero, Carmen Corciulo, Hailing Liu, Jin Zhang, Miguel Perez-Aso, Laura Picard, Tuere Wilder, Bruce Cronstein

**Affiliations:** 1 Division of Translational Medicine, Department of Medicine, NYU-Langone Medical Center, New York, New York, United States of America; 2 Bone and Joint Research Unit, IIS-Fundación Jiménez Díaz UAM, Madrid, Spain; 3 Department of Immunology and Rheumatology, LiHuili Hospital, Medical School of Ningbo University, Ningbo, China; University of Navarra School of Medicine and Center for Applied Medical Research (CIMA), SPAIN

## Abstract

**Background:**

Fibrosing diseases are a leading cause of morbidity and mortality worldwide and, therefore, there is a need for safe and effective antifibrotic therapies. Adenosine, generated extracellularly by the dephosphorylation of adenine nucleotides, ligates specific receptors which play a critical role in development of hepatic and dermal fibrosis. Results of recent clinical trials indicate that tenofovir, a widely used antiviral agent, reverses hepatic fibrosis/cirrhosis in patients with chronic hepatitis B infection. Belonging to the class of acyclic nucleoside phosphonates, tenofovir is an analogue of AMP. We tested the hypothesis that tenofovir has direct antifibrotic effects *in vivo* by interfering with adenosine pathways of fibrosis using two distinct models of adenosine and A2AR-mediated fibrosis.

**Methods:**

Thioacetamide (100mg/kg IP)-treated mice were treated with vehicle, or tenofovir (75mg/kg, SubQ) (n = 5–10). Bleomycin (0.25U, SubQ)-treated mice were treated with vehicle or tenofovir (75mg/kg, IP) (n = 5–10). Adenosine levels were determined by HPLC, and ATP release was quantitated as luciferase-dependent bioluminescence. Skin breaking strength was analysed and H&E and picrosirus red-stained slides were imaged. Pannexin-1expression was knocked down following retroviral-mediated expression of of Pannexin-1-specific or scrambled siRNA.

**Results:**

Treatment of mice with tenofovir diminished adenosine release from the skin of bleomycin-treated mice and the liver of thioacetamide-treated mice, models of diffuse skin fibrosis and hepatic cirrhosis, respectively. More importantly, tenofovir treatment diminished skin and liver fibrosis in these models. Tenofovir diminished extracellular adenosine concentrations by inhibiting, in a dose-dependent fashion, cellular ATP release but not in cells lacking Pannexin-1.

**Conclusions:**

These studies suggest that tenofovir, a widely used antiviral agent, could be useful in the treatment of fibrosing diseases.

## Introduction

Fibrosing diseases are a leading cause of morbidity and mortality worldwide [1]. Fibrosis, the excess accumulation of extracellular matrix (ECM), affects a variety of organs including, among others, the liver, lung, and skin. The structural components of the ECM, growth factors, cytokines, chemokines, and proteases, as well as central signaling cascades implicated in fibrogenesis and fibrolysis, are nearly identical in these different tissues [2]. The specific etiology of fibrosis in most organs is only incompletely understood nonetheless, an antifibrotic agent, pirfenidone was recently approved for the therapy of interstitial pulmonary fibrosis. No such agents have entered the clinic for the treatment of hepatic cirrhosis or skin fibrosis.

A growing body of work from different laboratories has implicated adenosine and its receptors in the pathogenesis of fibrosis in the lung, skin, liver and heart [3–5]. Adenosine, an extracellular nucleoside which is generated by the dephosphorylation of ATP, has been shown to both stimulate wound healing and promote fibrosis [6–8]. Cells transport ATP into the extracellular space via the specific transporter Pannexin-1 and other transporters and many cell types express nucleoside triphosphate phosphohydrolase (NTPP, CD39) and ecto-5’nucleotidase (CD73) on their surface which, sequentially convert ATP to AMP and adenosine. Extracellular adenosine acts at either A_2A_ (A2AR) or A_2B_ (A2BR) receptors, members of the large family of G protein coupled receptors, to directly stimulate fibroblast production of extracellular matrix and growth factors, leading to fibrosis [3]. It has been observed that Pannexin-1 and extracellular ATP are upstream regulators of Angiotensin II and TGF-β and trigger fibrosis in mechanical stretch-induced cardiac fibrosis [9]. Induced ischemia rapidly increases the glycosylation of Pannexin-1 and increases its trafficking to the plasma membrane. ATP release through Pannexin-1 channels is involved in cardiac fibrosis following myocardial infarction [10]. Bao et al. demonstrated that Panx-1 hemichannels provides the ligand for the adenosine A2A receptors that plays a role in the inhibitory signal at the back of the neutrophil, and inhibition of Panx-1 hemichannels blocked A2A receptor stimulation preventing cAMP accumulation and impairing migration and polarization of neutrophils [11]. Deletion of the enzymes involved in adenosine production (either CD73 or CD39 or both) prevents hepatic and skin fibrosis in murine models and deletion or blockade of either A2A or A2B receptors prevents hepatic, dermal or peritoneal fibrosis as well [12]. Moreover, mice lacking adenosine deaminase have a marked increase in extracellular adenosine and suffer from excess fibrosis in lung, skin and other organs and blockade or deletion of A2AR and A2BR prevents this fibrosis [13, 14]. Evidence from epidemiologic and case control studies also supports the role of adenosine receptors in fibrosis. The principal pharmacologic effect of the most widely consumed drug in the world, caffeine, is non-selective blockade of adenosine receptors. Caffeine is the major pharmacologic agent in coffee and consumption of coffee and caffeine (in soft drinks) is associated, in a dose-dependent fashion, with reduced risk of death and fibrosis from liver disease (Reviewed in [15]).

A recent report on the long-term follow-up of patients in a clinical trial of tenofovir for the treatment of hepatitis B suggests that tenofovir therapy, in contrast to other antiviral agents, reduces or reverses hepatic fibrosis [16]. Moreover, histologic data from a prospective study in HBV/HIV co-infected patients treated with tenofovir for just over 2 years demonstrated falling fibrosis scores, a trend not observed with other antivirals [17]. Once tenofovir is internalized into cells it is subsequently phosphorylated to the active metabolite, tenofovir diphosphate [18]. In a mechanism similar to that of NRTIs (Nucleoside reverse transcriptase inhibitors), tenofovir diphosphate competes with deoxyadenosine 5′-triphosphate, for incorporation into newly forming HIV DNA. Once incorporated, termination of the elongating DNA chain ensues, and DNA synthesis is interrupted [19]. These observations suggest that tenofovir,, might have direct anti-fibrotic effects.

Therefore, we tested the effect of tenofovir treatment on hepatic and dermal fibrosis in two different murine models and explored the mechanism by which tenofovir treatment blocks fibrosis in these models. We report here that tenofovir blocks Pannexin-1-mediated ATP release and prevents hepatic and dermal fibrosis in murine models in association with a marked reduction of adenosine release by those organs.

## Materials and methods

### Reagents

Bleomycin sulfate (Hospira) was purchased as 15 U lyophilized powder, and reconstituted according to the manufacturer’s guidelines with PBS at 2.5 U/mL. Thioacetamide (TAA) was purchased from Sigma. Tenofovir was purchased from Sequoia Research Products (UK). Tenofovir was dissolved by addition of 0.1 N NaOH to a final pH of 7.0 and filter sterilized (pore size, 0.2 μm) as previously described [20]. Tenofovir (10 mM stock solution) was also kindly provided by Gilead. Malachite green kit was purchased from Anaspec. Alkaline phosphatase colorimetric kit was purchased from Abcam. ATP determination kit (A22066) was purchased from Molecular Probes.

### Mice

*In vivo* experiments were conducted using C57BL/6 male mice that were up to 14 weeks old by the time of sacrifice. Animal care and experimental procedures were performed in accordance with the Institutional Animal Care and Use Committee of NYU School of Medicine and National Institutes of Health guidelines and were approved by the Institutional Animal Care and Use Committee of NYU School of Medicine. Experiments were performed in male mice, since we have previously observed that breaking tension and hydroxyproline content were more consistent in the skin of male mice than their female counterparts, in agreement with prior studies [21]. Mice had free access to food and drink and were housed in a facility with a 12h light/dark cycle with controled humidity and temperature. Animals were monitored daily to assess their health and behavior. Mice that were moribund, appeared hunched over or showed other signs of severe stress were euthanized. We carried out these experiments with 10 mice in each group for each experiment as with this number of mice we have 80% power to detect as little as a 25% difference with a standard deviation of 15% using a 2 sided analysis and a 95% confidence interval.

### Dose determination of antiviral therapy

Dose determination was performed via conversion of animal doses to human equivalent doses based on body surface area [22]. Consultation with Gilead Sciences supported the use of such doses in murine models.

### Experimental design: Antiviral therapy / administration of tenofovir to mice subjected to fibrosing agents

In an established model of adenosine-mediated skin injury [7], skin fibrosis was induced with bleomycin challenge (2.5 U/mL, 0.1 ml). Using a 27-gauge needle, 100 μL of filter-sterilized bleomycin (dose determined from pilot experiments) or PBS was injected subcutaneously into the upper back of the mice. Injections in the same site were carried out on alternate days for 18 days. C57BL/6 mice were treated with tenofovir (75 mg/kg) administered intraperitoneally daily, during the course of bleomycin treatment (n = 10 mice randomized for each treatment). To determine the specificity of tenofovir, other groups of animals were treated with lamivudine (3TC) (100mg/kg), another reverse transcriptase inhibitor which is an analogue of cytidine, administered subcutaneously (n = 10).

In an established model of adenosine-mediated liver injury, liver fibrosis was induced by thioacetamide (TAA [23]). Briefly, C57BL/6 mice were treated with the known hepatic fibrosis-inducing agent TAA (100 mg/kg in PBS, intraperitoneally, three times weekly for 8 weeks). Daily antiviral treatments with tenofovir (75 mg/kg) were administered subcutaneously, during the course of injury. Control mice received intraperitoneal injections of vehicle or appropriate drug treatment (n = 10 each treatment in a randomized procedure).

### Dermal morphometric measurements

Dermal morphometric measurements were performed as we have previously described [7, 24]. Following sacrifice the dorsal skin of the animals was shaved. Skin thickness was measured on 12 mm punch biopsies obtained from the back. Four different skin measurements were recorded per mouse, and an average of those recordings were made. Breaking strength of the skin was measured on the punch biopsies using a tensiometer (Mark-10 Series EG Digital Force Gauge, Mark-10 Corporation Copiague, NY). Forceps were clamped at the furthermost extremes of the biopsy sample and the point of maximal stress before tearing of the biopsy was recorded [7, 24].

### Histology, picrosirius red staining, and scar index determination

Paraffin sections were stained with hematoxylin and eosin (H&E) and picrosirius red as per the Puchtler method as previously described [24] or immunohistochemistry with antibodies α-SMA (Abcam, Cambridge, MA). Endogenous peroxidase activity was blocked with hydrogen peroxide. Tissue sections were digested with alkaline endopeptidase for 12 minutes at 42°C and primary antibodies were incubated overnight at room temperature. Primary antibody was detected with secondary anti-rabbit (Santa Cruz Biotechnology Inc, Santa Cruz) followed by Fast 3’3’-Diaminobenzidine (Sigma-Aldrich, MO USA) development, with hematoxylin nuclear counterstaining. Appropriate negative controls were included with the study sections. Slides were acquired using a Leica SCN400 Digital Slide Scanner. Cells were counted using the Slidepath Digital Image Hub (DIH). Sirius red staining was performed to evaluate the degree of fibrosis in the specimens, as established [25]. Birefringence pattern and hue were evaluated using polarized light microscopy to determine collagen density, pattern of deposition, and maturity. Normal tissues display fine collagen bundles with a yellow-green birefringence in a random pattern while soft tissue fibrosis results in formation of thicker, parallel collagen bundles with an orange-red birefringence [26]. The scar index is a quantitative analysis of fibrosis calculated by comparing the ratio of orange-red to yellow-green staining (higher values represent increased scarring) using SigmaScan Pro 5 software with a minimum of 3–6 sections per animal (n = 5 animals/group) [27, 28]. The same red or green color threshold was applied to sections of the same size for all the images. Results were expressed as red or green pixels/high power field (hpf) as well as a comprehensive scar index indicative of the degree of fibrosis.

Hepatic sections (5 cross-sections per liver) were harvested and stained with H&E or picrosirius red, as described previously [29]. Digitized photomicrographs (entire cross-sections at ×20; 5 sections per liver) were quantitated for total area of picrosirius red staining using SigmaScan Pro software v.5.0.0 (SPSS, Chicago, IL, USA). Color thresholds were applied for fibrotic and total hepatic area. Fibrosis was calculated as a percentage of total hepatic area and expressed as the average of randomly selected tissue sections from each liver (n = 9-10/experimental group); (n = 5/control group). Results were expressed as % fibrosis of total liver area.

### Toxin-induced adenosine release in mice

C57BL/6 mice were pretreated with vehicle or tenofovir (75 mg/kg) intraperitoneally or subcutaneously for the skin and liver experiments, respectively. Since the half-life of tenofovir is between 16–18 hours [18, 30, 31], it was imperative for levels to achieve steady state after 5 half-lives. After two days of pretreatment with antivirals, an injection of bleomycin (0.25 U, SubQ) or TAA (100mg/kg, intraperitoneally) was administered to induce an acute toxin-induced adenosine release response. Experiments were performed with at least 5 animals/group.

Twenty-four hours after the insult, 12 mm skin biopsies were taken and weighed so that tissue specimens of ~40 mg were analyzed. Briefly, skin biopsies were washed in PBS containing antibiotics (penicillin 200 U/L, streptomycin 200 ug/L, and fungizone 50 ug/L), cut into small pieces, and incubated in DMEM at 37°C, 5% CO_2_. In the animals treated with TAA the livers were harvested and specimens of ~40 mg were analyzed. Hepatic sections were washed in PBS containing antibiotics, as above, cut into small pieces and incubated in DMEM at 37°C, 5% CO_2_. After 4 hours of incubation, supernatants were collected for adenosine determination.

### Quantification of adenosine levels by high-pressure liquid chromatography (HPLC)

Adenosine was extracted from supernatants and quantitated by HPLC, as we have previously described. Proteins in the supernatants were denatured by addition of trichloroacetic acid (10% vol/vol). The trichloroacetic acid was extracted with freon-octylamine, and the supernatants were collected and stored at -80°C before analysis. The adenosine concentration of the supernatants was determined by reverse-phase HPLC [32]. Briefly, samples were applied to a CI8μBondapack column (Waters Chromatography Div., Milford, MA) and eluted with a 0–40%-linear gradient (formed over 60 min) of 0.01 M ammonium phosphate (pH 5.5) and methanol, with a 1.5 ml/min flow rate. Adenosine was identified by retention time and the characteristic UV absorbance spectrum, and the concentration was calculated by comparison to standards. In some experiments, the adenosine peak was digested by treatment with ADA (0.15 IU/ml, 30 min at 37°C) to confirm that the peak identified contained only adenosine. All samples were run in duplicate, and results were expressed as adenosine concentration (nM) per 12 mm biopsy, to normalize adenosine to the net weight of the sample.

### Quantification of dermal hydroxyproline content

Hydroxyproline content in tissue specimens was measured colorimetrically as described previously [33, 34]. Tissue specimens were hydrolyzed in 12 N HCl at 120°C. Hydrolysates were filtered and neutralized to pH 7 with NaOH (phenolphthalein added to ensure adequate neutralization). Samples were mixed with chloramine-T solution (1.4% chloramine-T, 10% *N*-propanol, and 80% citrate-acetate buffer). The mixture was incubated for 20 minutes at room temperature. Ehrlich’s solution (Dimethylamino Benzaldehyde P-Dmba, Isopropanol, Perchloric Acid) was added and the samples were incubated at 60°C for 20 minutes. Absorbance was measured at 560 nm. Standard curves (0 to 50 μg) were generated for each experiment using reagent hydroxyproline as a standard. Results were expressed as μg of hydroxyproline per mg of tissue.

### Permanent knockdown of cellular transporters and enzymes

RAW264.7 and HepG2 cells were used. To transfect shRNA, RAW264.7 cells and HepG2 cells (15,000 cells/ml) were plated and 24 hours later cells were incubated in the presence of Hexadimethrine Bromide (4μg/ml) and 10^8^ lentiviral transduction particles corresponding to mouse Pannexin-1 (SHCLNV-NM_019482), mouse Connexin-43 (SHCLNV-NM_010288.3), human Pannexin-1 (TRCN0000155349), human AK4 (TRCN000037554), human NME2 (TRCN000010110) and human scrambled (SHC002) with puromycin selection marker, for another 24 hours to allow transfection. Media was then replaced with αMEM containing puromycin (1μg/ml), changing the media every three days until selected clones formed. These clones were isolated and expanded until confluence. Scrambled shRNA (SHC002V) was used as control. Permanently silenced clones are kept in culture under puromycin selection.

### Determination of ATP Release into the supernate

Extracellular ATP was determined in RAW264.7, murine macrophages, and HepG2, human hepatocytes, cell lines using a bioluminescence assay in which ATP-dependent generation of light by recombinant firefly luciferase and its substrate D-luciferin was measured using an ATP determination kit (A22066) purchased from Molecular Probes and assays were performed according to manufacturer’s protocol. All assays were performed in triplicate.

### Enzyme activity assays

Extracellular phosphatase assays were performed using the malachite green assay, according to the manufacturer’s protocol. Briefly, AMP or ATP substrate was added at 100 μM to cells or recombinant enzyme. AOPCP, a known CD73 inhibitor, was used as a control. A phosphate free buffer containing 2 mM magnesium chloride, 120 mM sodium chloride, 5 mM potassium chloride, 10 mM glucose, and 20 mM HEPES was used. Results were obtained colorimetrically (λ = 620 nm) and compared to a standard curve of known phosphate concentrations. Alkaline phosphatase assays were performed using a colorimetric kit supplied by Abcam, according to the manufacturer’s protocol. Briefly, recombinant enzyme was pretreated with adefovir or tenofovir for 15 minutes, and the conversion of *p*-nitrophenyl phosphate to *p*-nitrophenol was determined colorimetrically (λ = 405 nm). Optical density was recorded and compared to a standard curve of known *p*-nitrophenol concentrations.

### Collagen-1 determination

NHDF cells grew to 70–80% confluence and medium was changed to edium with 0.2% BSA for at leat 16 hours. Cells were treated with Tenofovir 10 μM alone or in combination with CGS21680 10μM for 24 hours. P was harvested and quantified by BCA (Bio-Rad). 15ug proteins were loaded for SCS-page. After being blocked with 3% BSA in TBST, blots were incubated with anti-collagen I antibody (Southern Biotech) for 1 hour.

### Statistical analysis

Data were expressed as means±SD or ±SE wherever appropriate. Results were analyzed by Student’s *t* test or analysis of variance (ANOVA), as appropriate, with GraphPad software v.4.02 (GraphPad, San Diego, CA, USA). Values of *P*<0.05 were considered significant. For each statistical analysis, the experimental unit was a single animal, and statistical analysis was performed to compare groups of animals.

## Results

### Tenofovir protects against bleomycin-induced dermal fibrosis

**Fig 1:**
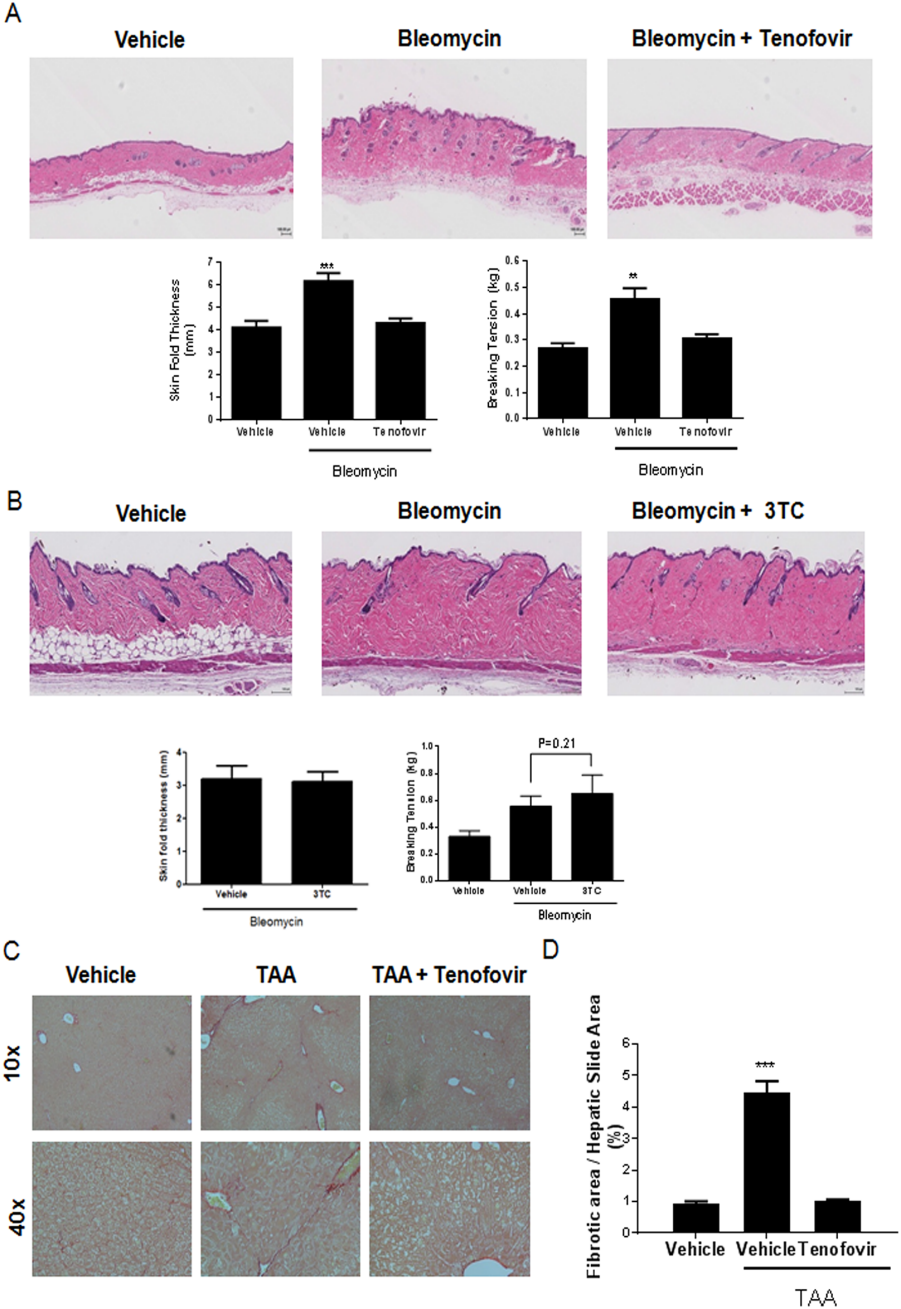
Tenofovir prevents liver and skin fibrosis in two models of adenosine-mediated injury. **A)** C57BL/6 mice treated with tenofovir therapy (intraperitoneal injections) are protected against bleomycin-induced dermal fibrosis. Bleomycin (0.25 U; SubQ injections, daily for 18 days) was used to induce fibrosis in C57BL/6 mice. Intraperitoneal tenofovir therapy (75 mg/kg) was administered during bleomycin challenge. **B)** C57BL/6 mice treated with 3TC therapy are not protected against bleomycin-induced dermal fibrosis. Images from representative hematoxylin and eosin stained slides are presented. Skin thickness measurements were performed as described in materials and methods. Breaking tension results were performed with a tensiometer and the recording at the point of maximal stress before tearing of the biopsy was recorded. **C)** Picrosirius red staining of fibrotic liver. TAA (100 mg/kg; intraperitoneal injections, alternate days for 8 weeks) was administered to induce fibrosis in C57BL/6 mice. Subcutaneous tenofovir therapy (75 mg/kg) was administered during TAA challenge. Paraffin-embedded liver tissue sections were stained in 1% Sirius red and collagen-positive sites were indicated in red. Digitized images (10x and 40x) are presented and show representative liver sections (n = 5–10). **D)** Quantification of picrosirius red staining was performed digitally using SigmaScan Pro v.5.0.0; data represent the percentage of total liver area stained by picrosirius red. Results are expressed as mean±SEM. Scale = 100 μm. **p<0.005, ***p<0.001 compared to vehicle (ANOVA).

To confirm that tenofovir had direct antifibrotic effects independent of its effects on viral infection we tested the effect of the drug in mice with bleomycin-induced dermal fibrosis, an established murine model of scleroderma. [35]. Tenofovir treatment markedly reduced skin thickness in bleomycin-treated mice compared with the skin of mice treated with bleomycin alone (4.3±0. mm versus 6.2± 0.3 mm, respectively, n = 5/group, p < 0.01) (Fig 1A). Moreover, tenofovir treatment reduced breaking tension in the bleomycin-treated mice (0.30±0.02 kg versus 0.46±0.04 kg, respectively [n = 5/group]; *P* < 0.01) (Fig 1A). When animals were treated with lamivudine, no significant changes were observed in either skin thickness (3.12±0.3 mm versus 3.2±0.4 mm respectively, n = 5/group, p = NS) or breaking tension (0.65±0.6 Kg versus 0.56.5±0.3 Kg, respectively n = 5/group; p = ns) (Fig 1B).

### Tenofovir diminishes skin alterations of bleomycin-treated mice

**Fig 2:**
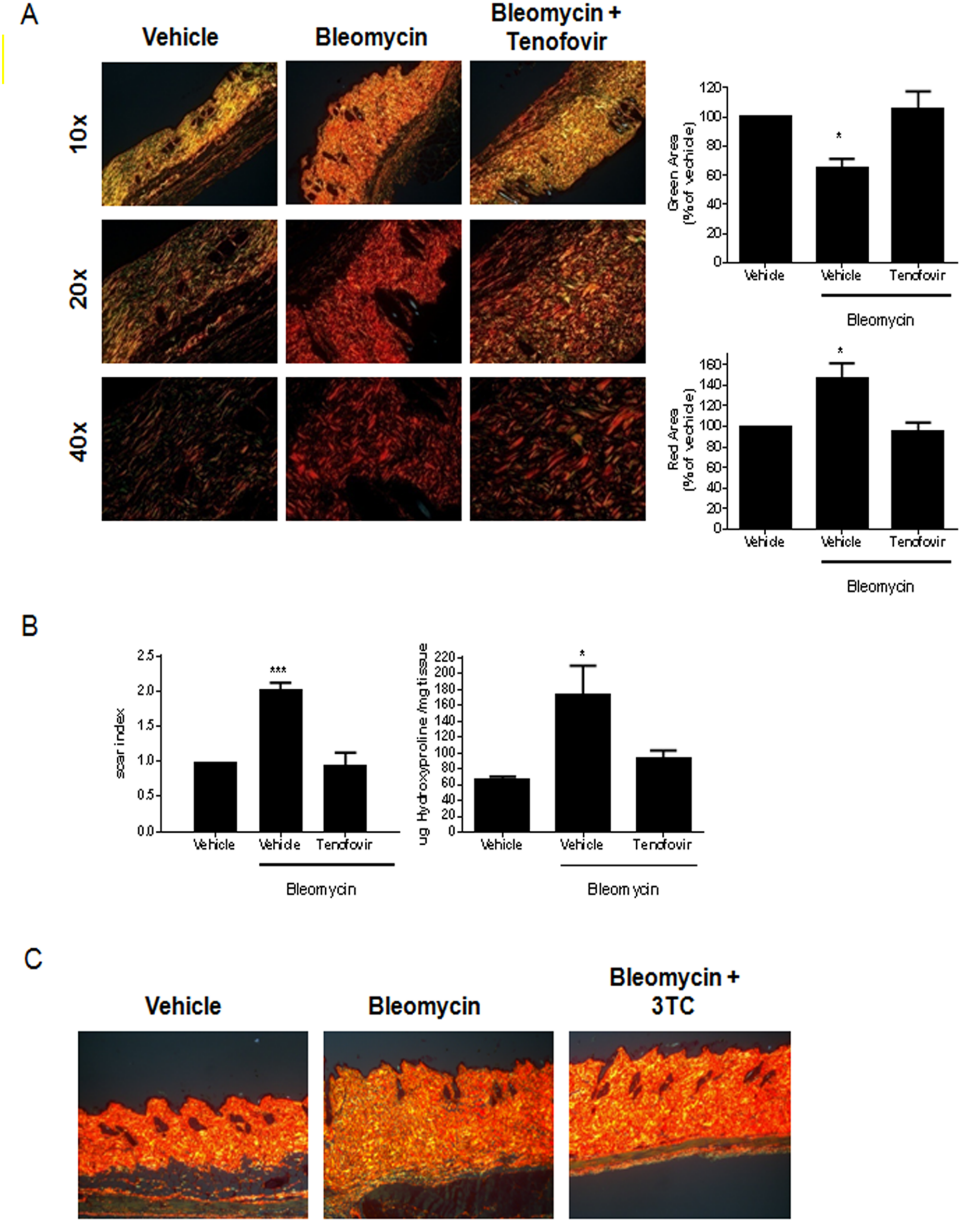
Tenofovir diminishes dense collagen fibrils. **A)** Representative Sirius red birefringence images are shown at 10x, 20x, and 40x original magnification. Orange-red is indicative of fibrosis; yellow-green is consistent with normal tissue. Biopsy site is near the site of induration 18 days after treatment. **B)** Quantitative analysis of red pixels/HPF and green pixels/HPF was performed with SigmaScan Pro software. Scar index measurements, quantitative analysis of fibrosis calculated by comparing the ratio of orange-red to yellow-green staining using SigmaScan Pro software. Hydroxyproline content is shown normalized to the mass (in mg) of the sample. **C)** Representative Sirius red birefringence images are shown at 10x original magnification. Orange-red is indicative of fibrosis; yellow-green is consistent with normal tissue. Biopsy site is near the site of induration 18 days after treatment. Hydroxyproline content is shown normalized to the mass (in mg) of the sample. Results are expressed as mean±SEM. Scale = 100 μm. *p<0.05, ***p<0.001 compared to vehicle (ANOVA).

To better evaluate the degree of fibrosis in the skin specimens, we further analyzed skin sections following staining with picrosirius red. Birefringence pattern and hue were evaluated using polarized light microscopy to determine collagen density, pattern of deposition and maturity. As shown in Fig 2A, biopsies from vehicle treated mice show a predominantly green birefringence whereas there was an increase in red birefringence with a decrease in green birefringence in bleomycin-treated mice (46±14% increased red birefringence and 36±6% decreased in green birefringence vs non-treated animals, p<0.05 respectively) [n = 5/group], a pattern typical of fibrosis (Fig 2B). Skin from tenofovir-treated mice demonstrated markedly diminished fibrotic changes; indeed, the skin resembled that of the vehicle-treated mice not treated with bleomycin (Fig 2A). Further quantitation of the red/green birefringence demonstrates the effect of bleomycin treatment on development of fibrosis and the reduction in fibrosis in the tenofovir-treated mice (Fig 2B). Bleomycin-treated mice had over a two-fold higher “scar index” than vehicle-treated mice, whereas tenofovir-treated mice did not suffer an increase in their scar index after bleomycin treatment (0.94±0.18 scar index versus 2.03±009, p<0.001, n = 5) (Fig 2B).

To further confirm the effect of tenofovir treatment on bleomycin-induced fibrosis we measured dermal hydroxyproline content as a reflection of total collagen content and degree of fibrosis (Fig 2B). As with the other measures of skin fibrosis, tenofovir treatment prevented the bleomycin-induced increase in dermal collagen. Hydroxyproline content was increased in bleomycin treated mice (173±37 μg hydroxyproline/mg tissue compared to 66±4 μg hydroxyproline/mg tissue for untreated mice, respectively, p<0.05) and levels were restored to nearly those of untreated mice in the presence of tenofovir (94±9 μg hydroxyproline/mg tissue, p = ns vs. untreated mice) [n = 5/group]. No reduction of bleomycin-induced fibrosis was observed when animals were treated with 3TC (Fig 2C).

**Fig 3:**
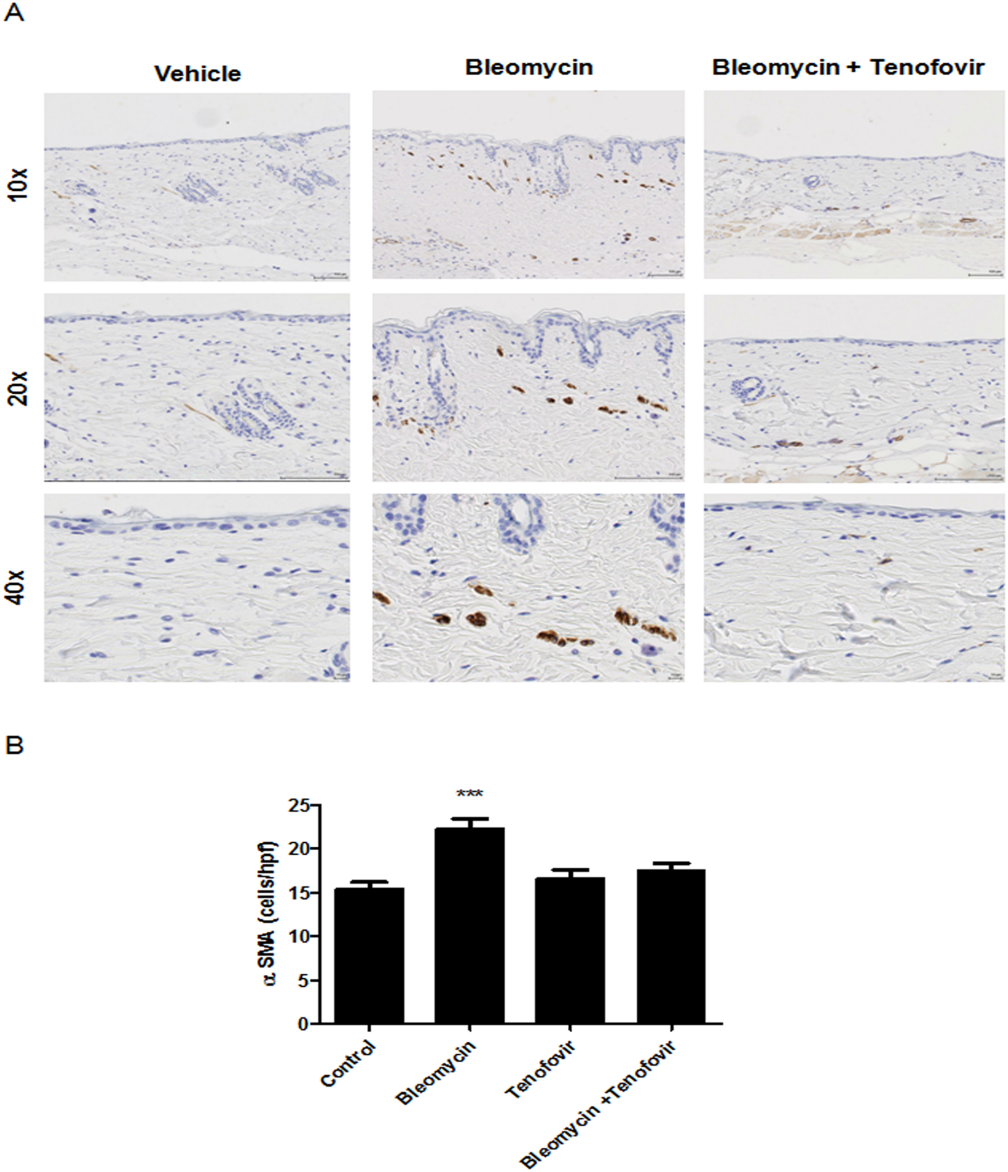
Tenofovir diminishes myofibroblasts in the skin of bleomycin-treated mice. **A)** Representative alpha-SMA immunostained images (100x, 200x, and 400x original magnification). Biopsy site is near the site of induration 18 days after treatment. **B)** Quantitative analysis of positive cells/HPF. Results are expressed as mean±SEM. Scale = 100 μm, ***p<0.001 compared to vehicle (ANOVA).

Consistent with the changes in skin matrix, bleomycin treatment stimulated a marked increase of the myofibroblast population (22±1 α-SMA^+^ cells versus 15±1 α-SMA^+^ cells for control, p<0.001, n = 5) that was reversed by treatment with tenofovir (17±1 α-SMA^+^ cells, p<0.001, n = 5) (Fig 3). Similar to collagen content and organization and skin thickening, myofibroblast accumulation was prevented by tenofovir 75mg/Kg (Fig 3).

### Tenofovir prevents fibrosis in a murine model of hepatic fibrosis

Tenofovir antiviral therapy is associated with continued improvement in liver histology in several reports [16, 17, 36]. To further confirm whether there was a direct anti-fibrotic effect of tenofovir we characterized the effects of this agent on hepatic fibrosis in an established murine model of hepatic fibrosis induced by thioacetamide (TAA, [37]). Consistent with our findings in the skin, in the TAA-treated mice there was clear formation of fibrous septa in the harvested livers (Fig 1C). Tenofovir treatment markedly reduced hepatic fibrosis in the TAA-treated mice (Fig 1C). Histomorphometric measurements (Fig 1D) were consistent with the histological appearance in the treatment groups; the TAA/tenofovir group of mice was protected from fibrosis as compared to the TAA/vehicle group (1.01±0.05% fibrotic area versus 4.44±0.37% for TAA, p<0.001, n = 10) (Fig 1C and 1D).

### Tenofovir does not affect either basal or A2A receptor-stimulated collagen 1 expression

Tenofovir has previously been reported to be a ligand for adenosine A1, A2B and A3, but not A2A receptors although the concentrations required are higher than are generally achieved *in vivo* [38, 39]. To determine whether adenosine receptor activation/inhibition by tenofovir could play a role in the diminished fibrosis we studied the effect of tenofovir on col1a1 expression by primary human dermal fibroblasts. Tenofovir neither inhibited basal collagen expression nor A2A receptor-stimulated (CGS21680, 1μM) by fibroblasts (S2 Fig).

### Tenofovir treatment of mice diminishes adenosine release from skin ex vivo

**Fig 4:**
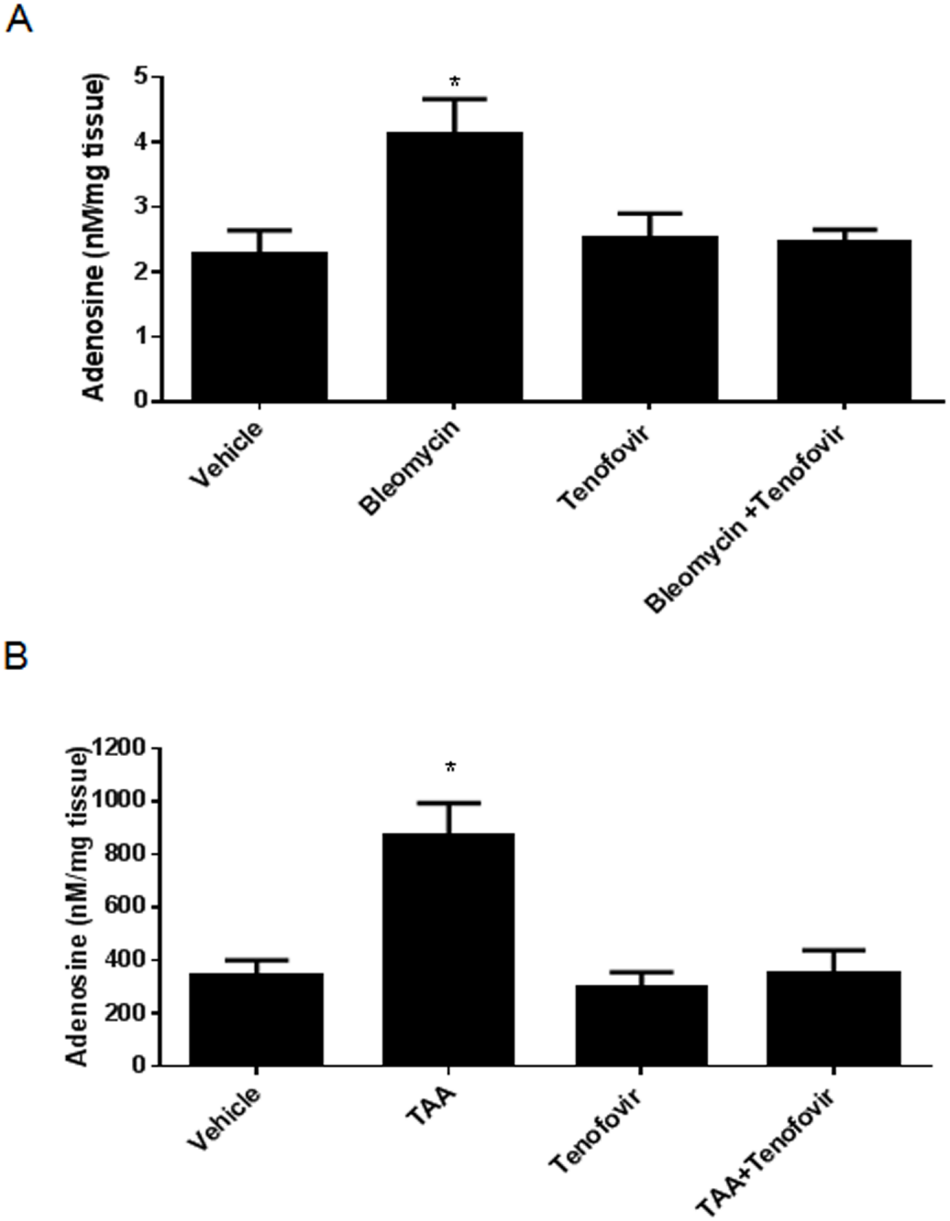
Tenofovir modulates adenosine levels. After two days of tenofovir pretreatment with (75 mg/kg)), an injection of bleomycin (0.25 U, SubQ) or TAA (100mg/kg, intraperitoneally) was administered to induce an acute toxin-induced adenosine release response. 24 hours later, animals were sacrificed and tissue was collected, adenosine extracted as described in methods and levels were measured by HPLC **A)** Bar graph of adenosine release in skin. **B)** Bar graph of adenosine release in liver. Results are expressed as mean±SEM. *p<0.05 compared to vehicle (ANOVA).

As we have previously reported that adenosine, generated by the action of CD39 and CD73, plays a critical role in development of both hepatic and dermal fibrosis in murine models of cirrhosis and scleroderma, respectively, [7, 23] we tested the hypothesis that tenofovir’s antifibrotic effects are mediated by inhibition of adenosine production. Mice were treated with tenofovir for two days and then bleomycin was administered, skin was harvested and cultured overnight *ex vivo*, adenosine levels in supernates were measured by HPLC, as described. Bleomycin treatment induced a two fold increase in adenosine release (4.13±0.53 nM/mg tissue compared to 2.26±0.37 nM/mg tissue for vehicle, p<0.005), an effect which was markedly reduced in skin from mice treated with tenofovir (2.47±0.18 nM/mg tissue, p = ns vs. vehicle) (Fig 4A) [n = 5/group]. Similarly, as we have previously reported, TAA treatment increases release of adenosine into the extracellular space by a CD73/CD39-mediated mechanism (872±120 nM/mg tissue for TAA compared to 345±55 nM/mg tissue for control, p<0.05) and treatment with tenofovir reduces adenosine release (297±58 nM/mg tissue for TAA, p = ns vs. vehicle treatment) (Fig 4B) [n = 5/group]. These findings are consistent with the central pathogenic role of adenosine in dermal and hepatic fibrosis in these models.

### Tenofovir does not increase adenosine release via inhibition of extracellular phosphatases

We have previously reported that the increase of adenosine release into the extracellular space that promotes fibrosis depends on CD39/CD73-mediated dephosphorylation of adenine nucleotides. Tenofovir is an AMP analogue that resembles substrates of CD39 and CD73. We therefore determined whether or not tenofovir inhibited dephosphorylation of adenine nucleotides (ATP or AMP). Tenofovir inhibited ATP dephosphorylation by 30% only at the highest concentration tested (100μM), a concentration far greater than can be achieved *în vivo* (S1 Fig). Similarly, neither tenofovir nor adefovir, a close structural analogue, inhibited alkaline phosphatase activity measured in HepG2 cells (S1 Fig). Thus, tenofovir does not alter extracellular adenosine levels by inhibiting conversion of extracellular adenine nucleotides to adenosine.

### Tenofovir inhibits ATP export via Pannexin-1

**Fig 5:**
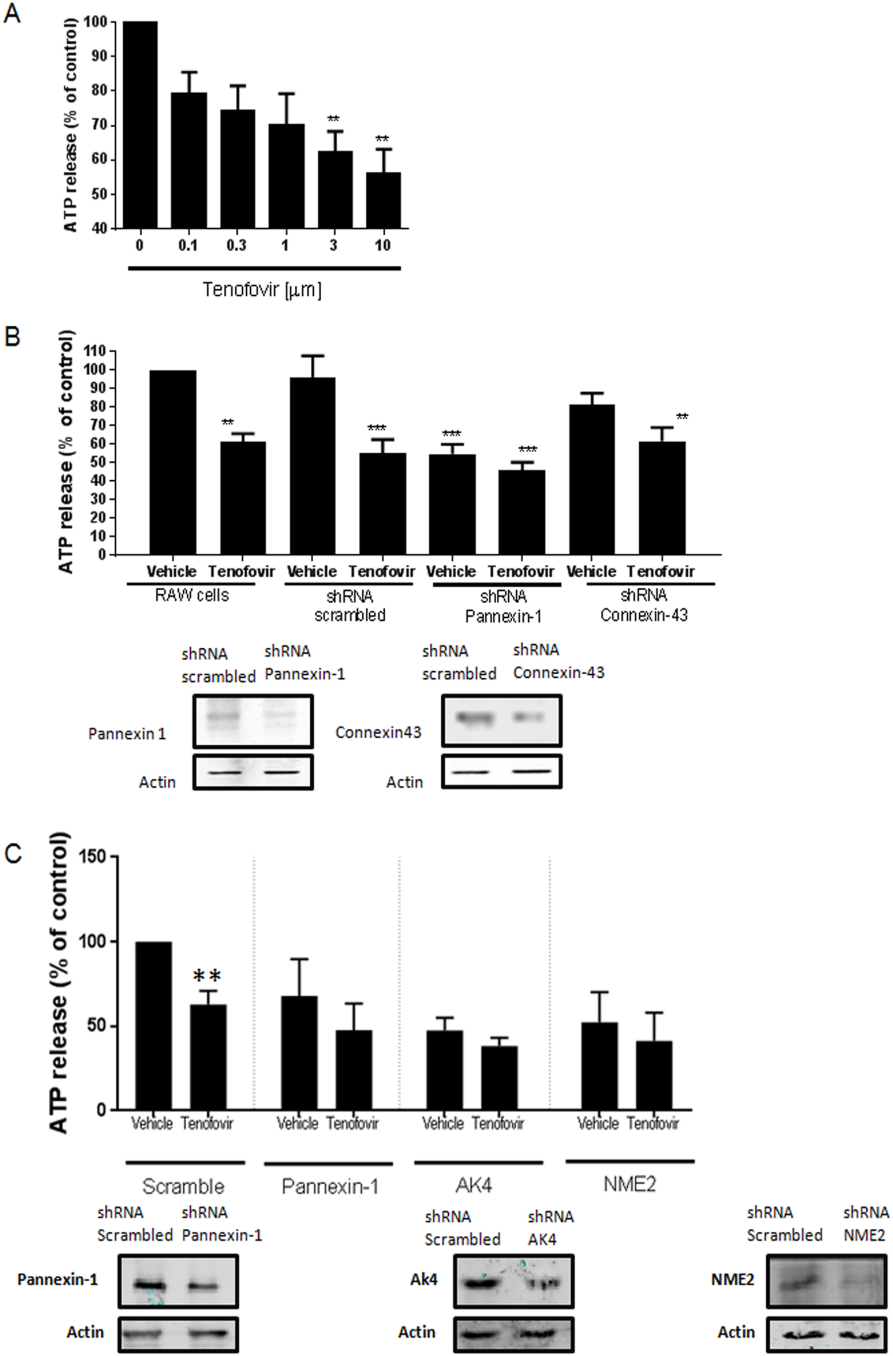
Tenofovir inhibits ATP export via Pannexin-1. **A)** *In vitro* ATP determination assays were performed with RAW264.7 cells according to manufacturer’s protocol. Tenofovir dose-response effects are shown. Data represents the mean results of 5 separate experiments carried out in duplicate. **B)** ATP determination assay was performed in the presence of Pannexin-1 or Connexin-43 knock-down (KD) RAW264.7 cells. Pannexin-1 and Connexin-43 knockdown protein expression in shRNA RAW264.7 cells. **C)** ATP determination assay was performed in the presence of Pannexin-1, AK4 or NME2 knock-down (KD) HepG2 cells. Pannexin-1, AK4 and NME2 knockdown protein expression in shRNA HepG2. Data is a representative experiment of two, performed in triplicate. Results are expressed as mean±SEM. **p<0.005, ***p<0.001 compared to vehicle in RAW264.7 or HepG2 cells (ANOVA).

To further investigate the mechanism by which tenofovir exerts its anti-viral potential, we explored the possibility that tenofovir inhibited ATP export from cells in both murine and human cells (Fig 5).

**Fig 6:**
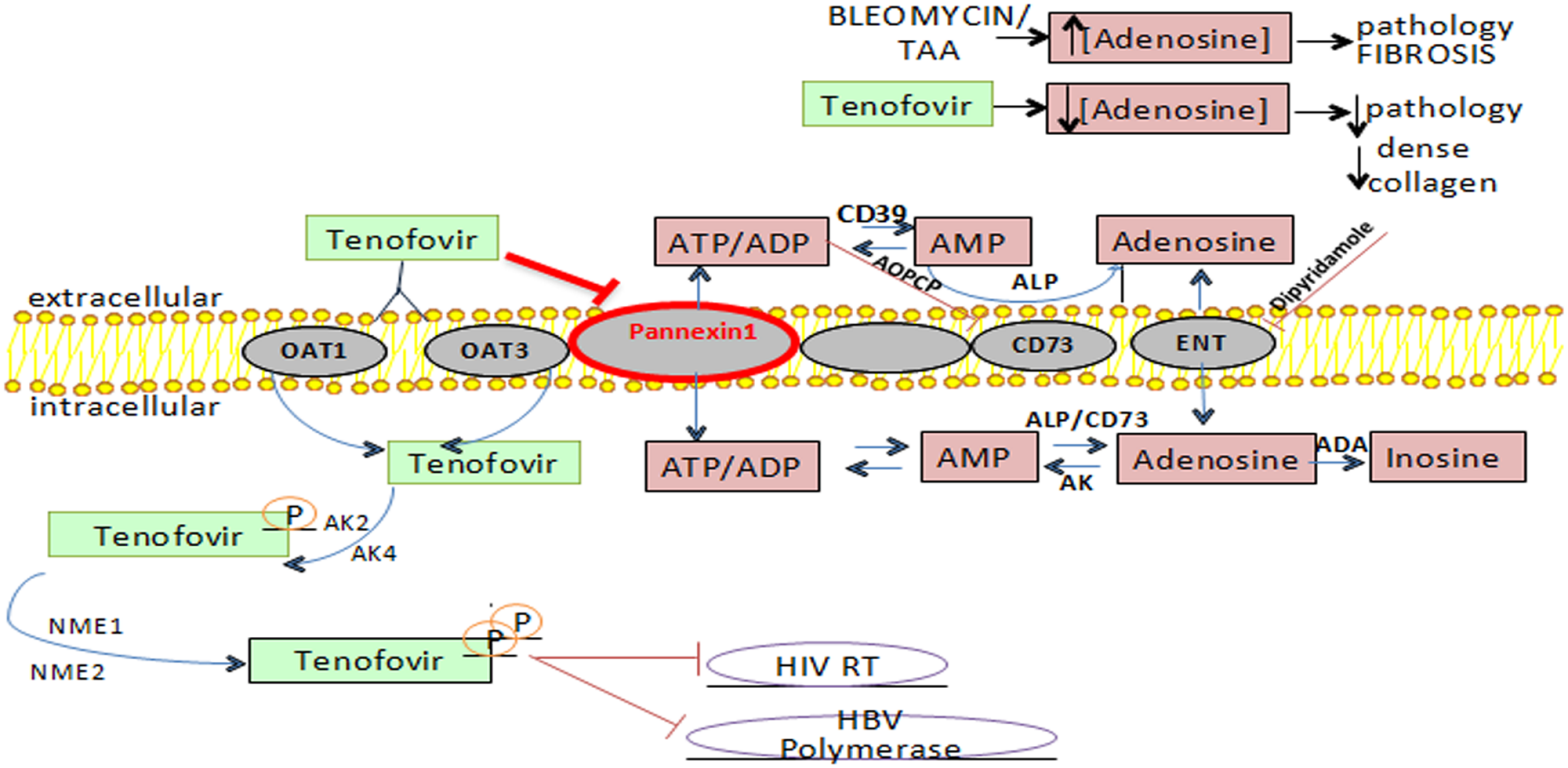
Model of tenofovir actions in the context of the adenosine axis. Tenofovir is phosphorylated. The enzymes catalyzing the phosphorylation steps are adenylate kinases (AK2 and AK4) and nucleotide diphosphate kinases (NME1 and NME2). Organic anion transporter 1 (OAT1) and organic anion transporter 3 (OAT3) are the major transporters involved in tenofovir clearance. In viral infected cells, tenofovir diphosphate inhibits the activity of HIV-1 reverse transcriptase by competing with its natural nucleotide counterpart deoxyadenosine 5'-triphosphate for incorporation into newly synthesized viral DNA. Once incorporated, it leads to termination of DNA elongation and stops further DNA synthesis. Major pathways are involved in adenosine metabolism. Adenosine is formed from its precursor ATP in both the intracellular and extracellular spaces. Intracellular adenosine is shunted into the extracellular space through membrane nucleoside transporters which also mediate uptake of adenosine from the extracellular fluid. The adenosine salvage enzyme adenosine kinase (AK) rephosphorylates adenosine to ATP while adenosine deaminase (ADA) deaminates adenosine to inosine. The extracellular formation of adenosine is the result of an enzymatic cascade consisting of the ectoenzymes, CD39, CD73 and alkaline phosphatase (ALP).

We observed that following overnight incubation, tenofovir inhibited ATP release from RAW264.7 cells in a dose dependent manner (IC50 = 2μM) (Fig 5A). Incubation of cells with tenofovir immediately before study of ATP release did not inhibit ATP release (not shown) indicating that tenofovir had to be taken up by cells and, presumably, converted to nucleotides to affect ATP export. Because Pannexin-1 and Connexin-43 have previously been reported to mediate ATP release into the extracellular space we determined the role of these proteins in ATP release and whether knockdown of these proteins altered ATP release and the capacity of tenofovir treatment to inhibit ATP release. Pannexin-1 and Connexin-43 protein expression was diminished by 77±1.4% and 44±10%, respectively by lentiviral expression of specific shRNA in RAW264.7 cells (compared to expression of scrambled shRNA). Tenofovir treatment inhibited ATP release by RAW264.7 cells infected with the lentivirus expressing scrambled shRNA (54.9±7.5% vs non-treated, p<0.001, n = 7) (Fig 5B). In RAW264.7 cells in which Pannexin-1, but not Connexin-43, expression was diminished there was a marked reduction in ATP release into the medium (46±5% decreased vs non-treated RAW264.7 cells, p<0.001, n = 7) (Fig 5B), as expected if Pannexin-1 is largely responsible for unstimulated ATP release in these cells. There was no effect of tenofovir treatment on ATP release by cells that lacked Pannexin-1 (46.22±1.19% vs non-treated, p<0.001, n = 7) (Fig 5B) whereas tenofovir reduced ATP release in Connexin-43-knockdown cells as well as cells expressing a scrambled shRNA or control cells (19±6% decrease for vehicle and 39±7% decrease for tenofovir vs non-treated RAW264.7 cells, p = ns and p<0.005 respectively) (Fig 5B). As shown in Fig 5C, tenofovir inhibited ATP release by HepG2 cells similar to RAW264.7 cells (37.1±8.1% decrease vs non-treated HepG2, p<0.005). When Pannexin-1, AK4 (Adenylate Kinase 4, the enzyme that catalyzes the interconversion of adenine nucleotides) and NME2 (Nucleoside diphosphate kinase B, a kinase involved in general homeostasis of cellular nucleoside triphosphates) were silenced by lentiviral expression of specific shRNA, no effect of tenofovir treatment on ATP release was observed (32.0±21.7% decrease for vehicle and 52.3±15.7% decrease for tenofovir in Pannexin-1 knockdown cells, p = ns; 52.5±7.4% decrease for vehicle and 61.7±4.9% decrease for tenofovir in AKA knockdown cells, p = ns; 47.5±17.7% decrease for vehicle and 58.7±16.7% decrease for tenofovir in NME2 knockdown cells vs non-treated HepG2, p = ns) (Fig 5C). These results are consistent with the hypothesis that uptake and phosphorylation of tenofovir diminishes Pannexin-1-mediated (but not Connexin-43-mediated) ATP transport into the extracellular fluid thereby diminishing the substrate for ectonucleotidases to convert to adenosine (Fig 6).

## Discussion

The success of antiviral therapies for viral hepatitis has established that, at least in liver, organ function can be markedly restored, with resolution of fibrosis, once the underlying source of injury is controlled. [40] Thus, data from patients demonstrates that tenofovir, but not other antiviral agents, reverses hepatic fibrosis/cirrhosis in patients with chronic hepatitis B [16]. In this study, we sought to characterize tenofovir’s actions in two murine models of fibrosis and report four novel findings: 1) Tenofovir’s impact on fibrosis is independent of its viral suppression capabilities; 2) Tenofovir prevents hepatic fibrosis development in a murine model of hepatotoxin-induced fibrosis; 3) Tenofovir protects against bleomycin-induced dermal fibrosis; 4) Tenofovir blocks Pannexin-1-mediated cellular ATP release leading to diminished adenosine levels in the extracellular space which diminishes A2AR-mediated fibrosis.

We have previously demonstrated that adenosine, generated by the extracellular dephosphorylation of adenine nucleotides, plays a central role in both skin and hepatic fibrosis due to bleomycin and thioacetamide, respectively. [41, 42] Either direct inhibition or deletion [7, 23] of adenosine A2AR or diminished adenosine production [41, 42] reduces fibrosis in these murine models. Results of epidemiologic and case-control studies provide further evidence that blockade of adenosine receptors can diminish hepatic fibrosis since consumption of coffee and other caffeine-containing drinks diminishes, in a dose-dependent fashion, the development of hepatic fibrosis and death from liver disease. The demonstration that tenofovir directly diminishes fibrosis and adenosine release suggests that tenofovir therapy might also be useful for the treatment of non-viral fibrosing conditions.

Tenofovir is a pro-drug; it is taken up by cells and converted to tenofovir polyphosphates which mediate the antiviral effects of the agent. The kinetics of tenofovir-mediated inhibition of ATP release is most consistent with the hypothesis that the intracellular accumulation of the drug, and likely its polyphosphated metabolites, mediates the effect of tenofovir on ATP release. Furthermore, although tenofovir itself is an analogue of AMP it does not interfere with the activity of ecto-5’nucleotidase. Thus, the capacity of tenofovir treatment to reduce adenosine release is due to reduction of the levels of precursors in the extracellular space where adenosine is generated from adenine nucleotides.

Previous studies demonstrate that A2AR directly stimulate collagen production and that they play an active role in the pathogenesis of hepatic and dermal fibrosis [7, 23, 43]. In prior studies, it was determined that A2AR occupancy directly stimulates collagen production in hepatic stellate cells [23, 44] and dermal fibroblasts [7], key mediators of liver and skin fibrosis, respectively. Moreover, A2AR regulates the quality of the collagen in the skin and, likely, the liver. [45] Thus A2AR clearly alter the balance of collagen I and collagen III (the abundance of which characterizes the matrix of scars) synthesis and accumulation. [46] The downstream signaling pathways for A2AR-mediated stimulation of collagen I and collagen III production also diverge. In human stellate cells and dermal fibroblasts A2AR regulate production of collagen I by a pathway dependent on protein kinase A, src, and ERK 1/2. In contrast A2AR regulates transcription and translation of collagen III via EPAC1/2 and p38MAPK. [44, 47].

Ligation of A2AR also regulates the expression of growth factors and cytokines known to play a role in fibrosis. Thus, A2AR occupancy promotes collagen production by stimulating CTGF production [43] and TGF-β. [48] A2AR-deficient mice have decreased levels of TGF-β and there are diminished levels of both CTGF and TGF-β in the skin of adenosine deaminase-deficient mice with dermal fibrosis after treatment with an A2AR antagonist [49].

Inflammatory cytokines play a role in hepatic and dermal fibrosis. IL-13 is a key mediator of tissue fibrosis and a potent stimulator and activator of TGF-β, as well as a direct stimulus for collagen production. [50] Adenosine stimulates IL-13 production and IL-13 reciprocally increases extracellular adenosine levels synergistically stimulating the development of fibrosis. [8, 51]

Moreover, Pannexin-1 and ATP release are involved in cardiac fibrosis and in adenosine A2A receptor activation in neutrophils [10, 11]. Here we demonstrate that tenofovir inhibits ATP release by Pannexin-1 hemichannels, and therefore diminishes adenosine levels. The demonstration that tenofovir treatment diminishes adenosine levels may also explain some of the toxic side effects of tenofovir treatment. There is a growing appreciation that patients with HIV disproportionately suffer from osteopenia and while there are many factors that may contribute to the loss of bone, tenofovir therapy is a significant risk factor. [52, 53] Previous studies have demonstrated that ecto-5’nucleotidase-deficient mice are osteopenic and that osteoblasts and their precursors produce adenosine which promotes bone formation. [54, 55] In addition, adenosine A2AR-deficient mice have diffuse osteopenia which results in part from a marked excess of osteoclasts and osteoclast-mediated bone turnover. [56–58] Thus, it is possible that diminished extracellular adenosine levels in the bone of patients treated with tenofovir could contribute to the development of osteopenia.

We found that knockdown of Pannexin-1 alone significantly reduces spontaneous ATP release from the murine macrophage cell line cells by an amount similar to that induced by tenofovir in control cells and tenofovir treatment of Pannexin-1 knockdown cells does not further inhibit ATP release. In contrast, Connexin-43 knockdown did not affect spontaneous ATP release by these cells under the conditions studied. In prior studies Connexin-43 required activation to promote ATP release (cf [59]) and it is likely that under the conditions in which the cells were cultured the contribution of Connexin-43 to spontaneous release of ATP is minimal. Moreover, the observation that tenofovir treatment did not affect ATP release by Pannexin-1 knockdown cells but significantly inhibited ATP release by Connexin-43 knockdown cells is most consistent with the hypothesis that tenofovir’s effect is selective for Pannexin-1.

Ecto-5’nucleotidase is critical for the production of extracellular adenosine and, with such an important regulatory role in the production of adenosine, this enzyme has become an appealing drug target with potential applications in the treatment of inflammation [60], chronic pain, [61] hypoxia, [62] and cancer. [63] Moreover, marked up-regulation of CD73 has been demonstrated in fibrotic diseases associated with chronic inflammation. Patients with chronic obstructive pulmonary disease or idiopathic lung fibrosis show markedly elevated pulmonary CD73 expression levels. [14] Conversely, genetically altered mice lacking CD73 have diminished fibrosis in skin and liver. [64–66] Thus, in addition to targeting adenosine production with tenofovir these studies suggest that targeting CD73 may also be suitable in modulating inflammation and fibrosis.

## Supporting information

S1 Fig Neither adefovir nor tenofovir affects extracellular phosphatases. **A-C)** Malachite green assay performed as per manufacturer’s protocol. AMP = 100 μM; 15 minute assay; (λ = 620 nm), Adefovir and Tenofovir range 1 nM-100 μM. **D-E))** Malachite green assay (Anaspec) was performed in HEPG2 cells treated with ATP or AMP substrate (100 μM), for 15 minutes, in the presence of pretreatment of adefovir or tenofovir. Phosphatase activity was expressed as fold change and normalized to phosphatase activity of cells treated with ATP alone. Inorganic phosphate release was a readout for phosphatase activity (λ = 620 nm). Activity was determined using a standard curve of known phosphate concentrations. **F)** Alkaline phosphate activity assay (Abcam) was performed according to the manufacturer’s protocol. Briefly, recombinant enzyme was pretreated with adefovir or tenofovir for 15 minutes, and the conversion of *p*-nitrophenyl phosphate to *p*-nitrophenol was determined colorimetrically (λ = 405 nm). Optical density was recorded and compared to a standard curve of known *p*-nitrophenol concentrations. Hu = human, tx = treatment.
(TIF)

S2 Fig Tenofovir does not affect either basal or A2A receptor-stimulated collagen 1 expression. Protein expression for Collagen I was determined after challenge NHDF in presence of CGS21680 10μM and Tenofovir 10μM for 24 hours. Data are expressed as mean ± sem. * P < 0.05, versus nonstimulated control (ANOVA).
(TIF)

S1 File NC3Rs ARRIVE guidelines checklist 2014. (DOC)

## Abbreviations

3TC: Lamivudine
AOPCP: α,β-Methylene-ADP
CD73: ecto-5’nucleotidase
ECM: extracellular matrix
TAA: Thioacetamide
