# Supplementary figures and images for: The antiviral drug tenofovir, an inhibitor of Pannexin-1-mediated ATP release, prevents liver and skin fibrosis by downregulating adenosine levels in the liver and skin
Source: PLoS One. 2017 Nov 16;12(11):e0188135. doi: 10.1371/journal.pone.0188135 (PMC5690602; doi:10.1371/journal.pone.0188135)

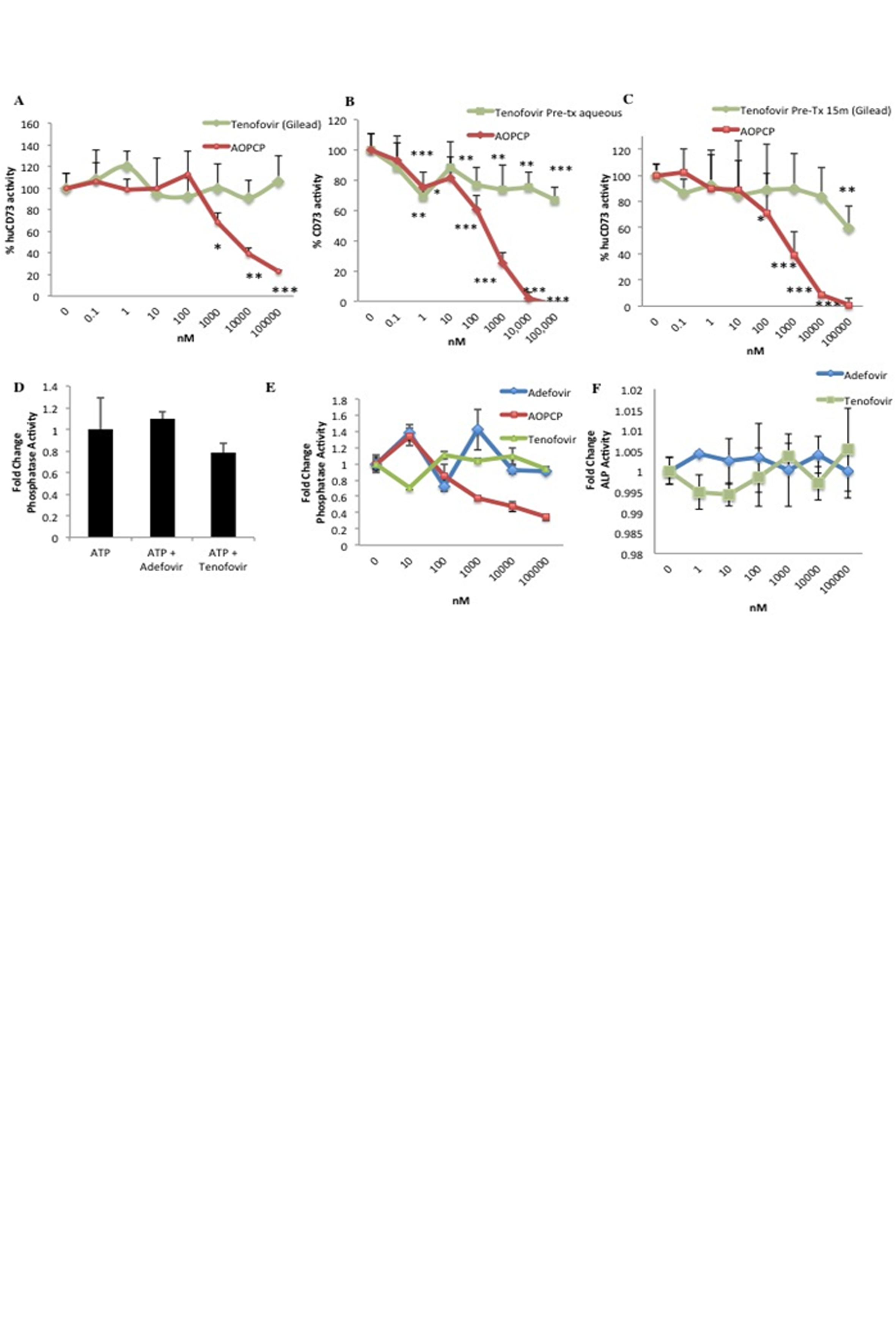

Supplement: S1 Fig — A-C) Malachite green assay performed as per manufacturer’s protocol. AMP = 100 μM; 15 minute assay; (λ = 620 nm), Adefovir and Tenofovir range 1 nM-100 μM. D-E)) Malachite green assay (Anaspec) was performed in HEPG2 cells treated with ATP or AMP substrate (100 μM), for 15 minutes, in the presence of pretreatment of adefovir or tenofovir. Phosphatase activity was expressed as fold change and normalized to phosphatase activity of cells treated with ATP alone. Inorganic phosphate release was a readout for phosphatase activity (λ = 620 nm). Activity was determined using a standard curve of known phosphate concentrations. F) Alkaline phosphate activity assay (Abcam) was performed according to the manufacturer’s protocol. Briefly, recombinant enzyme was pretreated with adefovir or tenofovir for 15 minutes, and the conversion of p-nitrophenyl phosphate to p-nitrophenol was determined colorimetrically (λ = 405 nm). Optical density was recorded and compared to a standard curve of known p-nitrophenol concentrations. Hu = human, tx = treatment. (TIF) [file pone.0188135.s001.tif]

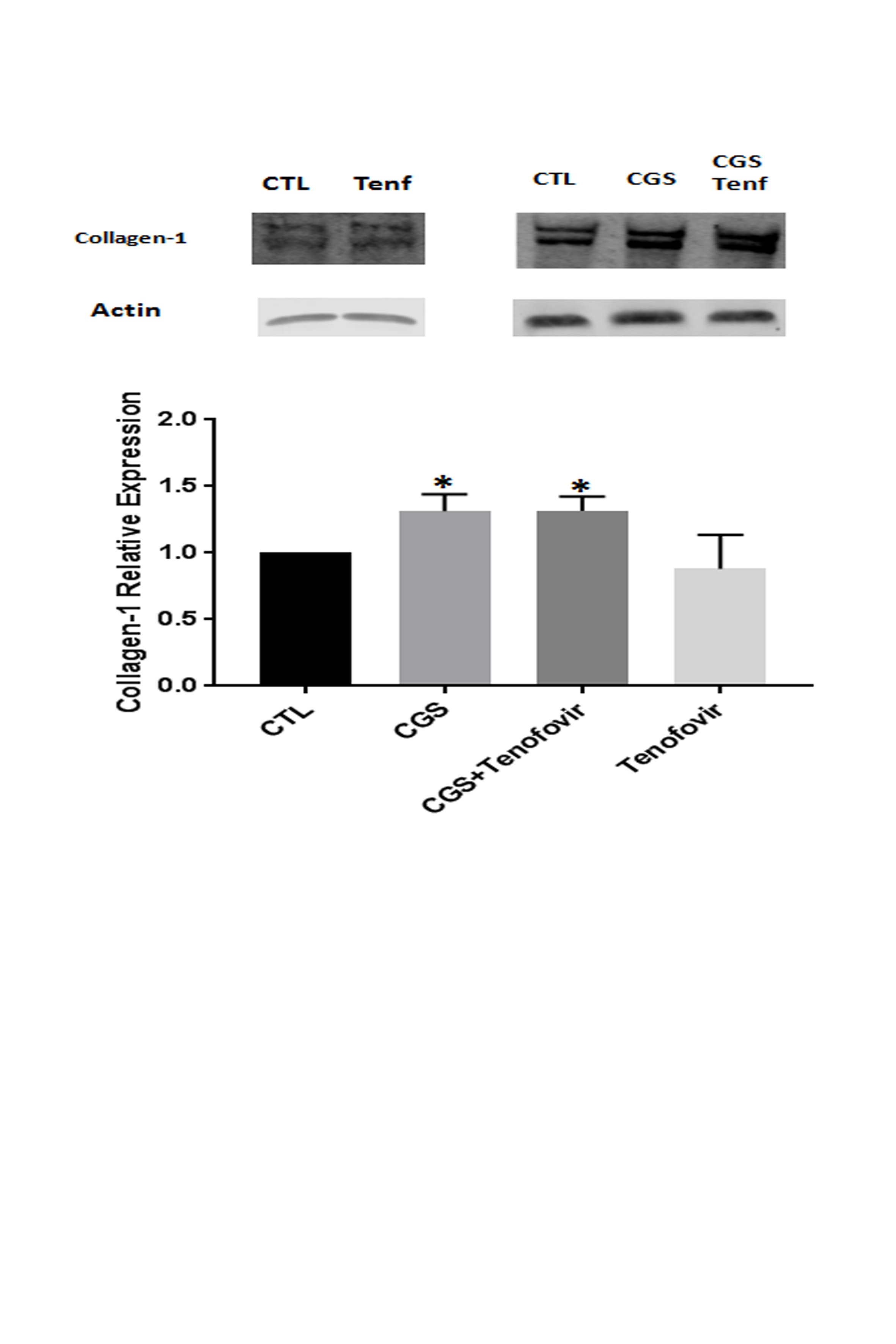

Supplement: S2 Fig — Protein expression for Collagen I was determined after challenge NHDF in presence of CGS21680 10μM and Tenofovir 10μM for 24 hours. Data are expressed as mean ± sem. * P < 0.05, versus nonstimulated control (ANOVA). (TIF) [file pone.0188135.s002.tif]
