# Supplementary material for: The antiviral drug tenofovir, an inhibitor of Pannexin-1-mediated ATP release, prevents liver and skin fibrosis by downregulating adenosine levels in the liver and skin
Source: PLoS One. 2017 Nov 16;12(11):e0188135. doi: 10.1371/journal.pone.0188135 (PMC5690602; doi:10.1371/journal.pone.0188135)

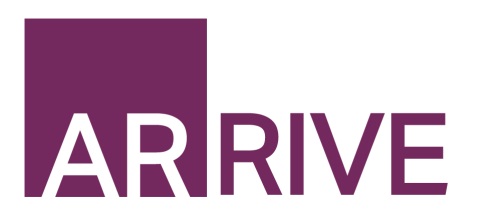
Etter.

The ARRIVE Guidelines Checklist

Animal Research: Reporting In Vivo Experiments

Carol Kilkenny^1^, William J Browne^2^, Innes C Cuthill^3^, Michael Emerson^4^ and Douglas G Altman^5^

*^1^The National Centre for the Replacement, Refinement and Reduction of Animals in Research, London, UK, ^2^School of Veterinary Science, University of Bristol, Bristol, UK, ^3^School of Biological Sciences, University of Bristol, Bristol, UK, ^4^National Heart and Lung Institute, Imperial College London, UK, ^5^Centre for Statistics in Medicine, University of Oxford, Oxford, UK.*

|  | ITEM | RECOMMENDATION | Section/ Paragraph |
| --- | --- | --- | --- |
| 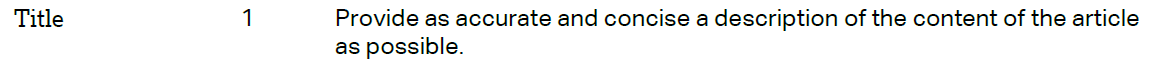 | | | Page 1 |
| 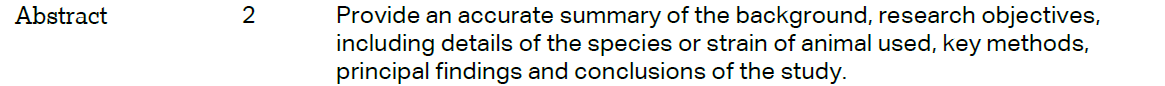 | | | Page 2 |
| INTRODUCTION | | |  |
| 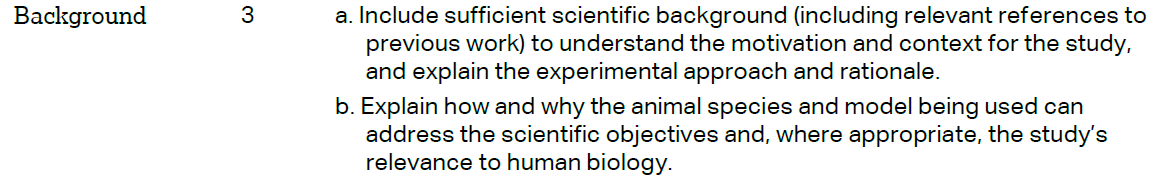 | | | Page 4 paragraph 1, Page 5 paragraph 2, Page 6 paragraph 2 |
| 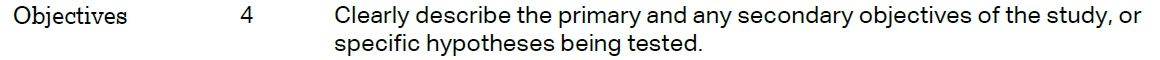 | | | Page 6 paragraph 2 |
| METHODS | | |  |
| 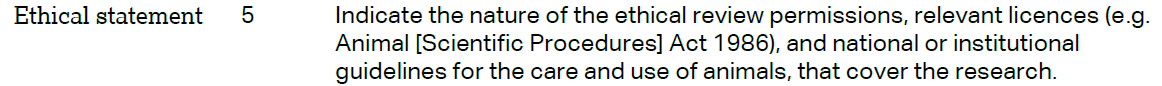 | | | Page 7, section Mice |
| 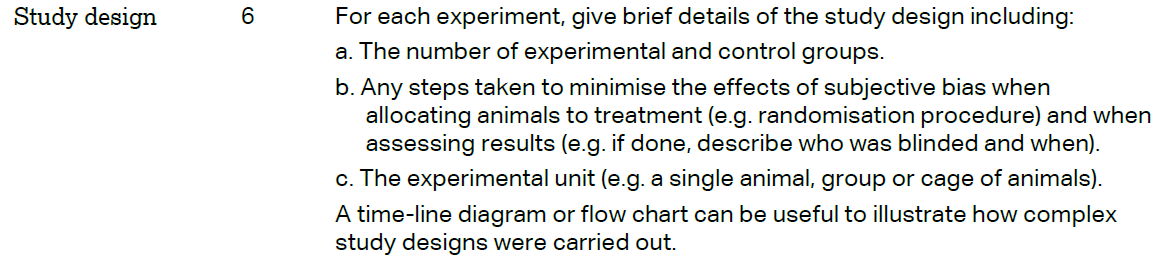 | | | Page 7, last paragraph, page 8 paragraph 1, |
| 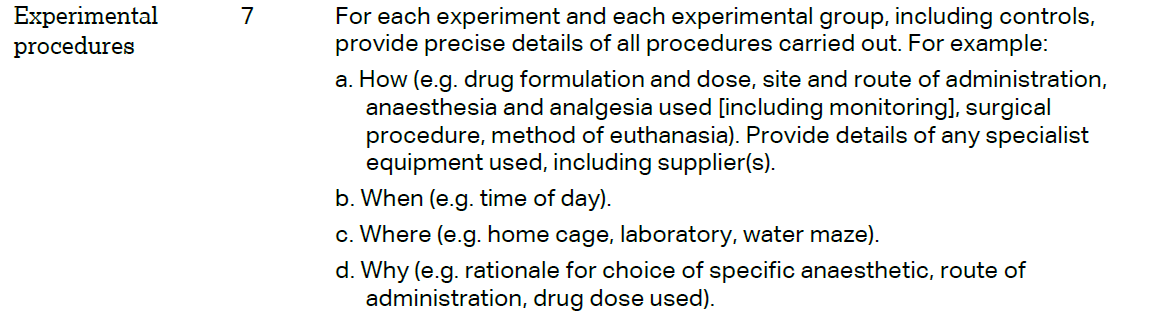 | | | Page 7 and 8 section Experimental design: antiviral therapy / administration of tenofovir to mice subjected to fibrosing agents |
| 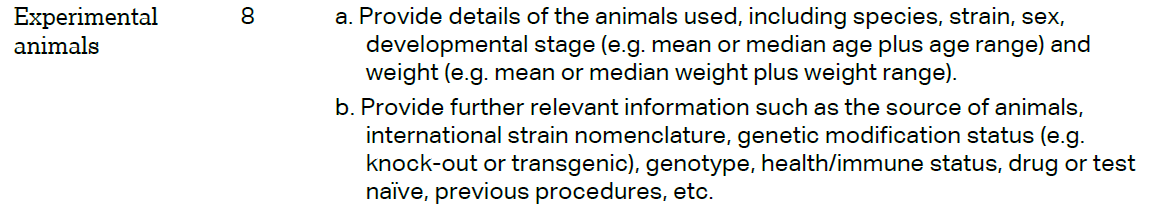 | | | Page 7, section Mice |

The ARRIVE guidelines. Originally published in *PLoS Biology*, June 2010^1^

| 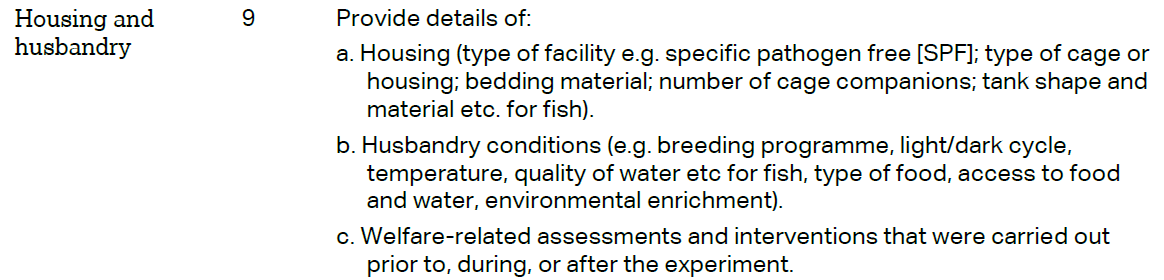 | Page 7, section Mice | |
| --- | --- | --- |
| 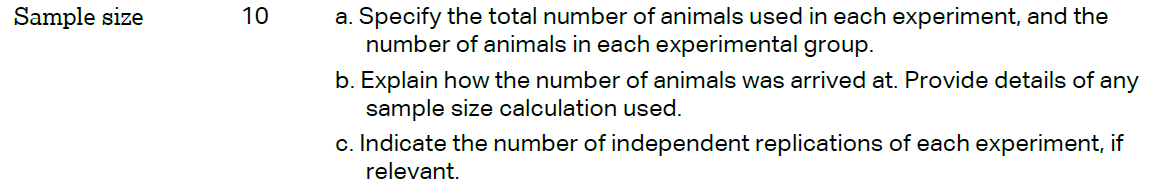 | Page 7, section Mice | |
| 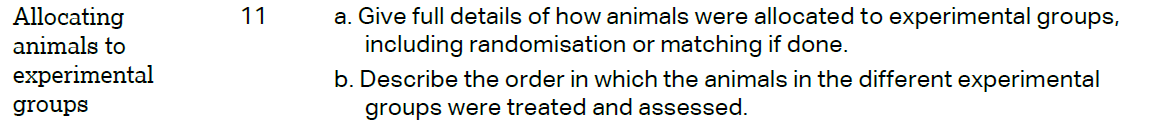 | Page 8 section Experimental design: antiviral therapy / administration of tenofovir to mice subjected to fibrosing agents | |
| 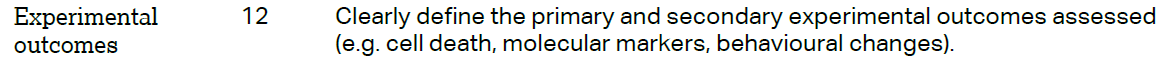 | Page 7, section Mice | |
| 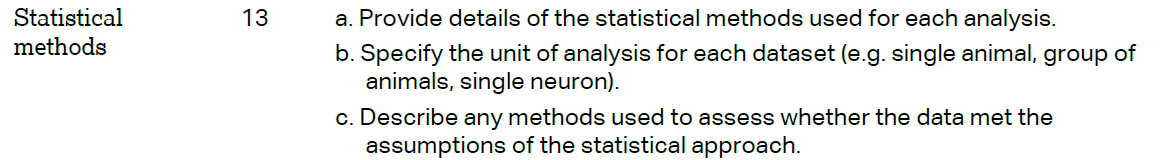 | Page 13-14 Statistical analysis section | |
| RESULTS |  | |
| 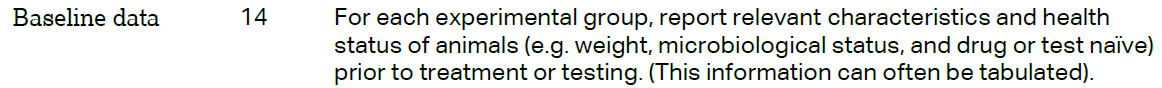 | Page 7 section Mice | |
| 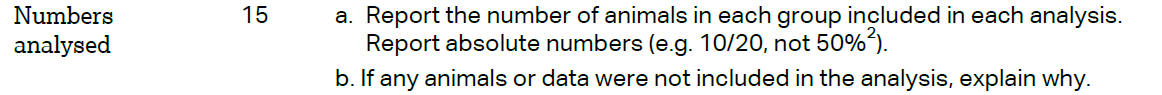 | Page 14 to page 17 | |
| 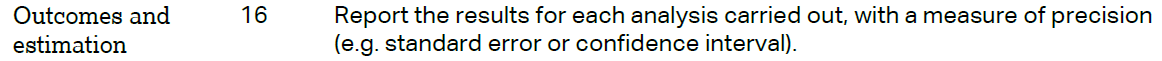 | Page 14 to page 17 | |
| 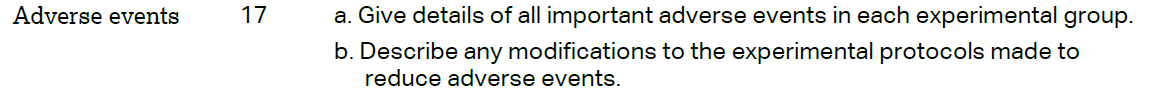 | Page 7 section Mice | |
| DISCUSSION |  | |
| 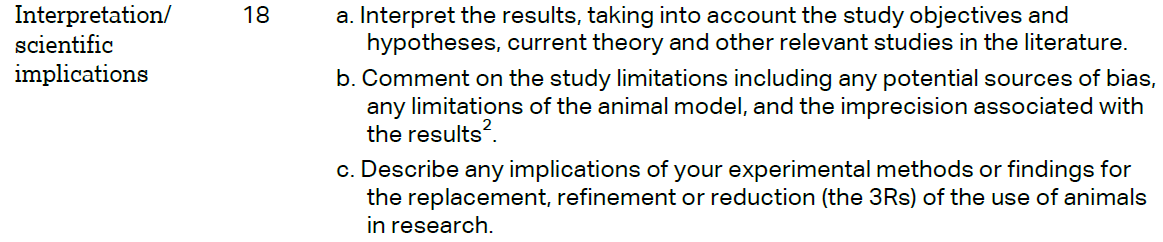 | Page 19 to page 22 | |
| 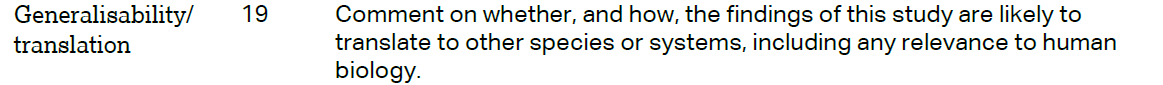 | Page 19 to page 22 | |
| 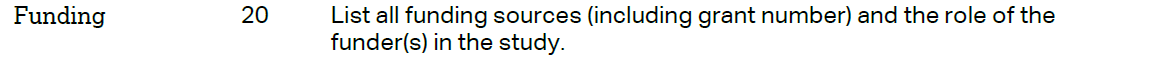 | Page 23 Acknoledgemts section |  |


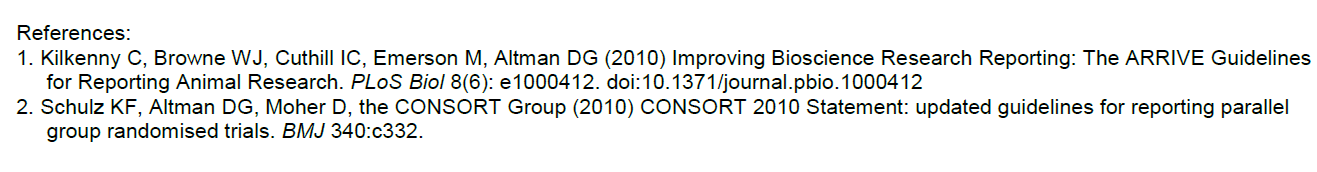

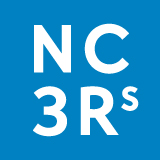

Supplement: S1 File — (DOC) [file pone.0188135.s003.doc]
